# Many chronological aging clocks can be found throughout the epigenome: Implications for quantifying biological aging

**DOI:** 10.1111/acel.13492

**Published:** 2021-10-16

**Authors:** Hunter L. Porter, Chase A. Brown, Xiavan Roopnarinesingh, Cory B. Giles, Constantin Georgescu, Willard M. Freeman, Jonathan D. Wren

**Affiliations:** ^1^ Oklahoma Medical Research Foundation Oklahoma OK USA; ^2^ University of Oklahoma Health Sciences Center Oklahoma OK USA; ^3^ Oklahoma Center for Geroscience and Healthy Brain Aging Oklahoma OK USA

**Keywords:** Aging, bioinformatics, epigenetic clocks, epigenetics

## Abstract

Epigenetic alterations are a hallmark of aging and age‐related diseases. Computational models using DNA methylation data can create “epigenetic clocks” which are proposed to reflect “biological” aging. Thus, it is important to understand the relationship between predictive clock sites and aging biology. To do this, we examined over 450,000 methylation sites from 9,699 samples. We found ~20% of the measured genomic cytosines can be used to make many different epigenetic clocks whose age prediction performance surpasses that of telomere length. Of these predictive sites, the average methylation change over a lifetime was small (~1.5%) and these sites were under‐represented in canonical regions of epigenetic regulation. There was only a weak association between “accelerated” epigenetic aging and disease. We also compare tissue‐specific and pan‐tissue clock performance. This is critical to applying clocks both to new sample sets in basic research, as well as understanding if clinically available tissues will be feasible samples to evaluate “epigenetic aging” in unavailable tissues (e.g., brain). Despite the reproducible and accurate age predictions from DNA methylation data, these findings suggest they may have limited utility as currently designed in understanding the molecular biology of aging and may not be suitable as surrogate endpoints in studies of anti‐aging interventions. Purpose‐built clocks for specific tissues age ranges or phenotypes may perform better for their specific purpose. However, if purpose‐built clocks are necessary for meaningful predictions, then the utility of clocks and their application in the field needs to be considered in that context.

## Graphical abstract

Epigenetic clocks show promise as a biomarker of aging. Our study identifies components of epigenetic clocks critical to understanding their uses and where improvements are still needed. We identified a parabolic trajectory of methylation aging and the extent to which epigenetic clocks are robust to removing the most age‐predictive loci. These data shape our understanding of epigenetic age acceleration and what epigenetic clocks can tell us about the biology of aging.

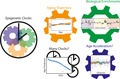

## 1 INTRODUCTION

Predicting age from molecular data has been a long‐standing interest in aging research because it implies that we can identify the correlative/causal factors behind aging. By extension, if molecular changes associated with age‐related diseases can be identified, hypotheses about potentially effective interventions that extend biological life and healthspan can be made. The first molecular predictors of chronological age included telomere length and p16INK4A levels(Tsygankov et al., 2009) but, recently, “epigenetic clocks” have supplanted them in accuracy and precision(Horvath, 2013), as well as their ability to predict all‐cause mortality risk (Perna et al., 2016). Since these original reports on mortality risk, studies have increasingly used epigenetic clocks to evaluate interventions that may extend lifespan in both mice(Wang et al., 2017) and humans(Fahy et al., 2019). Epigenetic clocks that can quantify a “biological age” that is distinct from chronological age, could have a profound impact on aging research. However, such clocks depend upon understanding the degree to which epigenetic changes at clock sites reflect altered physiological states, versus solely the passage of time which, outside of forensic applications, is already known.

Epigenetic clocks are multivariate machine learning models that predict age using methylation levels from a set of genomic cytosine sites. A downside of the machine learning approach inherent to epigenetic clocks is that the behavior of individual sites across time, as well as the biological regulation and impact, is often obscured. In addition, the ability to build equivalent performing clocks from the same data through small algorithmic differences does not provide a prioritization for biological relevance of the clock loci. Indeed, any machine learning method exhibits a “black box” nature which can obscure the biological relationship between methylation and variables of interest. Based upon the training data, which includes chronological age and CpG methylation levels at specific loci in a set of samples, algorithmic “learning” weighs each data point and combination of data points to choose sites whose methylation pattern best linearly correlates with chronological age. Unlike telomere shortening, which has a fairly straightforward biological impact to understand, there is no obvious biological interpretation of the relevance of sites used by epigenetic clocks to predict age. Prior studies demonstrate enrichment for some functionally relevant features, such as polycomb repressor targets (Horvath, 2013) and even that clocks can be trained on data from just these targets (Yang et al., 2016). This, however, leaves open the question of whether epigenetic marks in these regions are causative of aging, or if “reversing” epigenetic aging is sufficient to ameliorate age‐associated dysfunctions. Nonetheless, the field has extrapolated that epigenetic clock predicted ages that are older or younger than the individual's chronological age may represent “accelerated” and “decelerated” biological aging (Fahy et al., 2019; Fransquet et al., 2019; Horvath & Raj, 2018). Thus, it is becoming increasingly important to better understand how epigenetic clocks work and the biological relevance of the sites used.

Epigenetic clocks have been built using a wide variety of different genomic loci to predict age. Clocks can vary from three sites (Weidner et al., 2014) to any number, with the most popular epigenetic clock built by Horvath using 353 sites (Horvath, 2013). These clocks do have some overlap in sites chosen, but they are predominantly composed of unique genomic sites (Horvath & Raj, 2018). The fact that different clocks can arrive at similar accuracy using many different loci in the genome raises at least two questions. First, just how much of one's epigenome changes with age and to what extent? To this end, prior work has identified many potentially age‐related loci in blood (Slieker et al., 2016), but the extent of changes in pan‐tissue models and outside of array data is unknown. Second, if multiple regions are equally predictive, then do they have anything in common biologically?

A key biological insight from many of the epigenetic clocks is found in their ability to identify loci that molecularly “age” similarly across tissues, that is, pan‐tissue clocks. Epigenetic clocks select loci that predict age regardless of their tissue source, implying some pan‐tissue mechanism is driving epigenetic aging. This is despite the established role of methylation in determining cell types and large methylome differences between tissues. In order to be used as an effective biomarker of clinical aging, epigenetic clocks must be able to predict aging using tissues that can be sampled pre‐mortem (e.g., blood) and reflect changes in tissues affected by aging (e.g., brain). If these age‐predictive epigenetic changes are not common across tissues, we must find a way to translate within individuals if clocks are to be used to evaluate interventions that prevent age‐related diseases. Since epigenetic age “acceleration” is predictive of all‐cause mortality using just blood samples(Perna et al., 2016), we hypothesize that the age‐predictive methylation changes will be similar across tissues. However, prior work studying the relationship between pan‐tissue clocks and neurodegenerative disease has found the potential for “false positives” coming from these pan‐tissue signals that warrant even further scrutiny (Shireby et al., 2020).

While methylation differences between tissues are large, we found epigenetic clock sites change by only 1.5% on average between young (<35 years of age) and aged (>65 years of age) samples. Applications of epigenetic clocks in the field often use pan‐tissue clocks to evaluate tissue‐specific “biological aging”(Levine et al., 2016; McKinney et al., 2018; Ward‐Caviness et al., 2016). Tissue‐specific clocks may be necessary for clock models to predict age with loci that will also correlate with age‐related dysfunction in that tissue. Because the process of aging comes with predictable phenotypes and increased risk of age‐related diseases, when methylation changes are hypothesized to reflect “biological aging,” they should have a relationship to the cellular aging of the examined tissues. This leads us to two hypotheses. First, that samples from individuals with age‐related diseases would be epigenetically “older” than control counterparts. Second, age‐predictive loci should be consistent when training clocks across the lifespan and tissue types.

Elastic net regression, used to create epigenetic clocks, is predicated on selecting the best set of unique predictors, not necessarily all age‐predictive sites in the genome. Epigenetic clocks using other genomic sites have been developed for cellular senescence (Lowe et al., 2016), obesity (Sargent, 2015), cancer (Zheng et al., 2016), and even spawned their own theories of aging (Horvath & Raj, 2018). Taken together, all of these clocks suggest methylation at a large number of sites in the genome can be indicative of health and disease and that there are commonalities across tissues. However, the actual age‐related variation in methylation at clock loci is very small (<5%). Thus, the detected methylation changes with age could be attributable to changes in cellular abundance of a relatively rare cell subtype in examined tissues (e.g., infiltrating macrophages) rather than an epigenetic change in the resident cells of a tissue. Alternatively, there could be a common form of age‐related cellular change across cell types and tissues (e.g., cellular senescence). Or worse, the correlation could be spurious. Quoting Calude and Longo(Calude, 2017), "One of the main ideas supporting data analytics is that a series of correlations will continue or iterate similarly along the chosen parameter (recurrence). If, for example, time is the main parameter [...], then the correlation will extend into the future by iterating a similar “distance”, typically, between the chosen observables."Algorithmic identification of potentially interesting patterns within large datasets holds great potential for advancing scientific understanding. It is, however, not a substitute for it. Mathematically, the larger a dataset, the more arbitrary correlations it will contain (Calude, 2017). Thus, once identified, it is incumbent upon us to identify how (and if) such patterns inform our current understanding. To assay the breadth of sites in the genome that are associated with age, potential mechanisms that could explain the ability to generate a clock model, potential interacting partners of DNA methylation‐modifying enzymes that influence changing methylation with age, and understand what pathways may be affected by age‐related methylation changes, we conducted a large‐scale analysis of human methylation data using the Illumina 450k methylation array platform, for which we collected 9,699 samples of adults (aged 25+) with age and tissue descriptions using our automated sample annotation approach (Giles et al., 2017).

## 2 RESULTS

### 2.1 Epigenetic changes with age are small in magnitude across the lifespan

**FIGURE 1:**
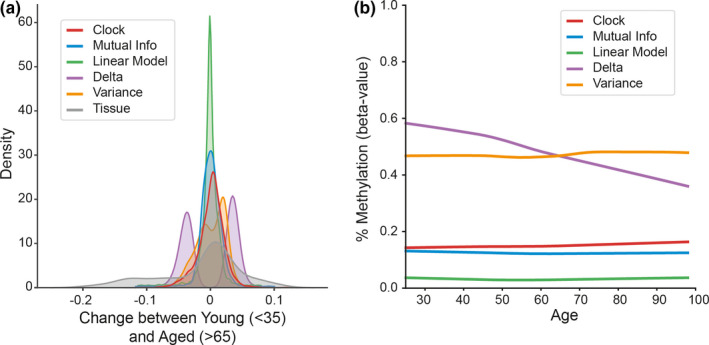
Average lifespan change of age‐predictive loci is small. Age‐related loci were selected by different methods: epigenetic clocks (red), linear models (green), top 10% mutual information (blue), top 10% greatest change in mean (“delta,” purple) or variance (orange). (a) Histogram of age‐related changes in methylation over the lifespan (beta values from 0 to 1.0), averaged across samples. An example tissue difference of blood versus brain is also presented (gray). Aside from sites with the greatest mean difference with age (purple) and between tissues (gray), age‐related sites exhibit small magnitude changes over the lifespan. (b) The most informative individual locus by each method is displayed as a lowess‐smoothed fit over age

The magnitude of epigenetic clock site (ECS) methylation changes across the lifespan is significantly smaller than the largest age‐related changes within non‐clock sites, and smaller still than known differences in DNA methylation between tissues (Figure 1). Looking at age‐related sites by a variety of metrics, we see that age‐predictive sites by linear regression and non‐linear mutual information regression follow the same pattern of small magnitude changes found in clock sites. We then analyze the highest weighted locus by each metric (e.g., epigenetic clock site in red) (Figure 1b). Even the most “age‐related” locus, as identified by multiple methods, shows a small magnitude difference over the lifespan. This finding leads to many questions. How do such small age‐related changes occur consistently across individuals and tissues composed of multiple cell types? Are these small differences occurring in a restricted set of loci, or are there large numbers of these sites from which a variety of clocks can be constructed? Perhaps methylation values reach an asymptote and regress toward the young values?

### 2.2 Age predictions from epigenetic clocks are replicable, but use different loci

A logical starting point in determining the number of possible ECSs is to recreate Horvath's original clock. We independently collected as much of the original data that was publicly available from NCBI Gene Expression Omnibus (GEO), obtaining ~75% of the training data. We selected a set of loci whose methylation state can predict age using the same preprocessing, including imputation, normalization, and elastic net modeling.

**FIGURE 2:**
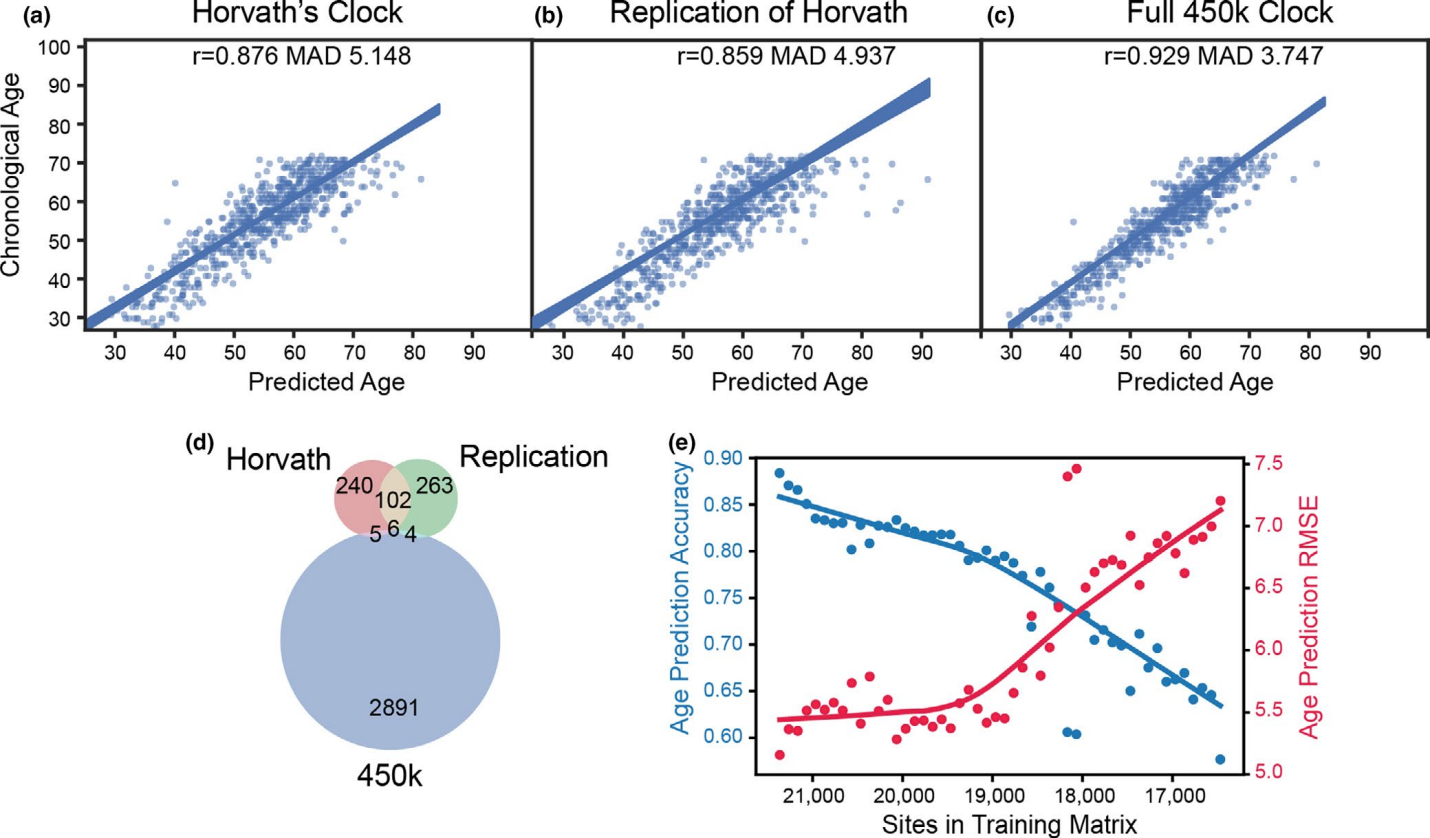
Different epigenetic clocks select different sites but perform similarly. (a) Horvath's original model sites (353) showing predicted vs actual age on a single test set of blood samples (GSE42861) (b) The model based on replication of Horvath's method produced predicted vs actual age on GSE42861 using all available training data from Horvath's original paper (Horvath, 2013). The model performed slightly worse with the smaller training set, and also selected fewer sites (252). (c) A model trained using all age‐annotated 450k data with sample labels from ALE(Giles et al., 2017), was tested on the same set of blood samples (GSE42861). This model performed better than the original Horvath clock, but also had access to a much larger set of loci for training and selected a larger set of loci (2906) to predict age. (d) Venn diagram of clock loci used in each model. The models selected different sites despite similar prediction quality with the only variables being different training samples and a different random seed. (e) Pearson correlation between predicted and actual age (blue) and root mean squared error (RMSE, red) by number of loci remaining in training set. Each point represents a new model trained with all loci used by previous models removed

We replicated the results of the original Horvath model (Figure 2a, 2b). We also trained a model on 9699 samples of Illumina 450k methylation array data deposited in NCBI GEO (Figure 2C), which allowed us to use data from more sites (~450,000) than the 21,369 sites used in the Horvath report. We only included samples from experiments with over 100 samples, as we found that including smaller experiments quickly diminishes clock performance. Our full 450k model outperformed the original Horvath model, but this is expected as it had access to more features in both the training and test sets. While some data from Horvath's original paper were unavailable and/or lacked age data that were publicly available, we demonstrated roughly equivalent performance across the three models with mainly different sets of sites in each model (Figure 2d).

Because clock models trained on almost identical data can select different loci as the most predictive set and perform equivalently, we examined how many loci could be used as clock sites via an iterative “knockout” approach. After using elastic net regression to identify the most informative loci, these sites were then removed and new clock models trained on the remaining sites. We fit 5 new clock models after every removal and removed any locus used by any of the 5 clocks. By iteratively removing the most predictive sites every round, subsequent models should become increasingly hindered in their performance. Interestingly, rather than a rapid depletion of predicted performance, we saw a gradual linear decrease in prediction accuracy (as defined by Pearson correlation coefficient between predicted and actual age, Figure 2e) and rise in model error (Figure 2e) until about 20% of sites have been removed from the training data. These data illustrate the breadth of CpGs that are strong age predictors from the sites measured by the Illumina 450k microarray.

### 2.3 Pan‐tissue epigenetic clocks fail to identify tissue‐specific epigenetic aging

DNA methylation has known roles in specifying tissue identity (Koh & Rao, 2013; Macaluso & Giordano, 2004) via differentially suppressing and activating specific regions of the genome in a cell type‐specific manner. Our own data and previous reports (Horvath, 2013; Lowe et al., 2016; Thompson et al., 2018) on pan‐tissue epigenetic clocks demonstrate that chronological age‐predictive models work equally well across most tissues when trained on many tissues. However, clocks trained on blood alone have poor performance at predicting tissue‐specific impairments, such as cognitive decline(Starnawska et al., 2017).

This raises the question of whether pan‐tissue clocks either still capture enough tissue‐specific/relevant changes by inclusion of multiple tissues in the training set, or if pan‐tissue clocks are missing tissue‐relevant changes, and potentially sacrificing insight into tissue‐specific aging and disease.

**FIGURE 3:**
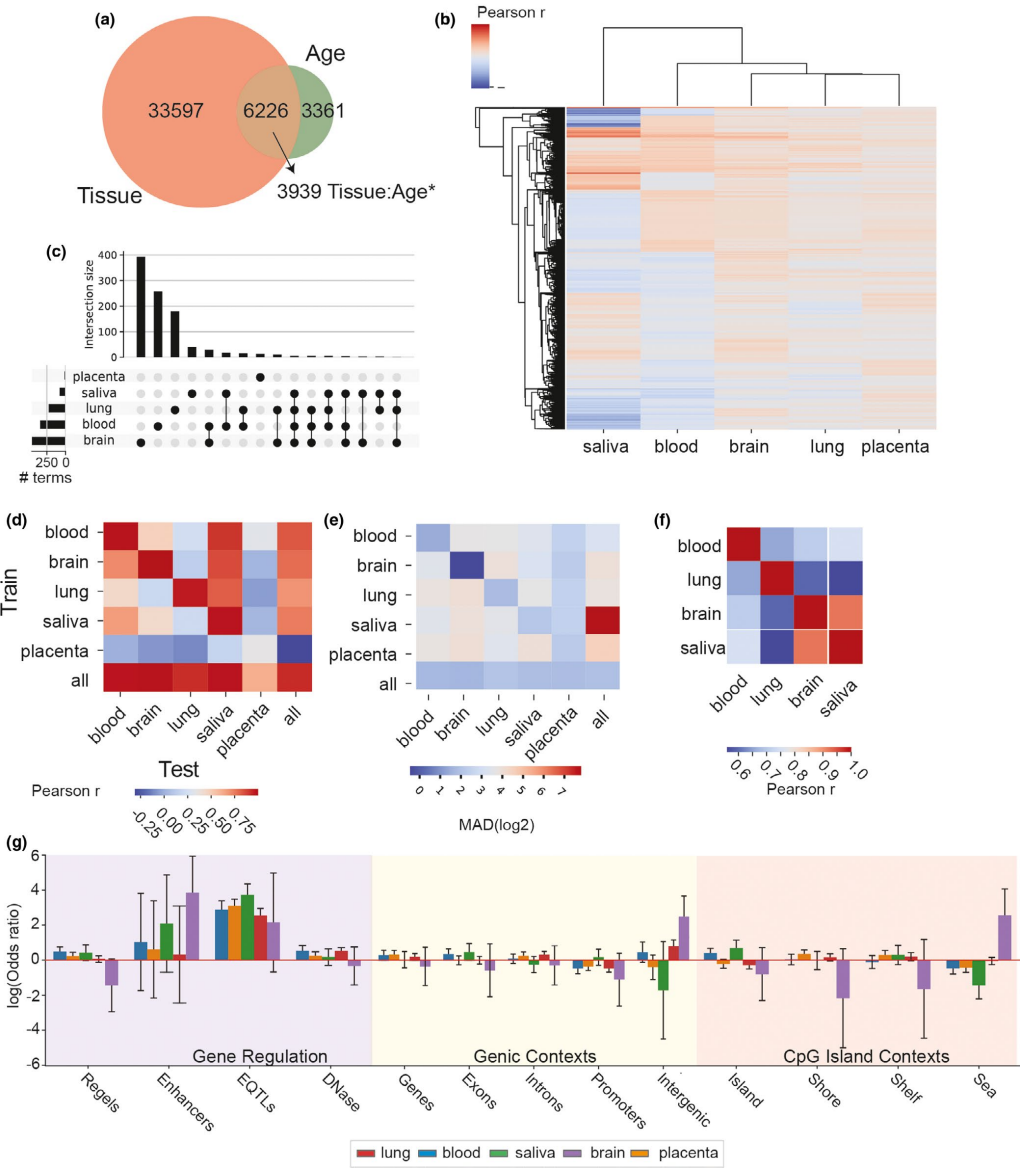
Modeling tissue‐specific aging methylation changes. (a) Venn diagram of the overlap between methylated loci that significantly differ between tissues and those that change with age in the 450k data. Multiple testing correction was done using a Benjamini–Hochberg 10% FDR correction. (b) Clustering of all sites with a significant age association by their per‐tissue aging correlation coefficients. Coloration indicates methylation‐age correlation coefficients for that specific tissue. (c) UpSet plot showing the number of loci in each trained clock and their combinatorial overlaps. (d and e) Matrix of correlation between predicted and actual age from epigenetic clocks trained and tested on a per‐tissue basis. Epigenetic clocks were trained on the tissues in the Y‐axis and tested on the tissues in the X‐axis. Model correlations between predicted and actual age shown in (d) while median absolute deviation is shown in (e). (f) Matrix of correlations between clock site coefficients common between at least 3 of 5 clocks trained on specific tissues. The placenta clock did not have any sites common to at least 3 other models G. Bar plots of log odds ratios showing enrichment\depletion of loci from clocks with different training tissues with respect to regulatory features, genic features, and CpG islands, corrected for the coverage of 450k methylation array

To look for tissue‐specific age association, we used an ordinary least squares regression to model the effects of age, tissue, and their interactions. 39823 loci were significantly (q<0.05) associated with tissue, while 9587 were associated with age. Of the 6226 sites with significant main effects of both tissue and age, 3939 had a significant interaction effect, suggesting a tissue‐specific aging change. The rest of the age‐associated sites had tissue‐independent effects (as defined by non‐significant tissue:age interactions), suggesting common age‐related changes across tissues (Figure 3a, c). Looking closer at the loci with significant age main effects, the tissue‐specific aging interactions are weak (Figure 3b). This suggests that there is some common process across tissues that causes these changes regardless of whether it is a regulated program or a systemic form of epigenomic entropy. Dysregulations of epigenetic machinery have been implicated as the driver of the relationship between DNA methylation and aging(Bell et al., 2019), but our prior work in mouse found no changes in the direct methylation machinery with aging(Hadad et al., 2016).

Observing that most loci with a main effect of age were similar across tissues (except for saliva) was surprising in light of the strong relationship between methylation and tissue identity. This leads us to question whether elastic net would identify ideal pan‐tissue predictors as the best sites to predict age even in a single tissue, or if they would be too weak of a signal compared to tissue‐specific signals. When we trained clocks on data from only one tissue at a time, they perform markedly worse on predicting age in other tissues (Figure 3d and e). This indicates that the tissue‐specific signals are in fact strong enough to drown out the non‐tissue‐specific signals, which are more abundant. Notably, saliva sample ages were uniquely well‐predicted by all other tissue models, but poor predictors of other tissue ages. When comparing the coefficients of loci identified by elastic net for multiple tissues, we do see moderate associations (*r* > 0.5) for all tissues—indicating that some loci are being chosen that covary even by tissue‐specific models (Figure 3f). The loci chosen by these tissue‐specific clocks are largely uniformly distributed across genomic features, but are notably enriched near GTEX (Lonsdale et al., 2013) eQTLs. Perhaps the enrichment of these loci near regions where point mutations are sufficient to affect disease‐related gene expression is indicative of a potential biological link, but determining whether these changes are causal, compensatory, or silent in the aging process will require further studies in epigenome editing/manipulation.

### 2.4 Age‐predictive loci depend upon which ages are used to train and test the clock

**FIGURE 4:**
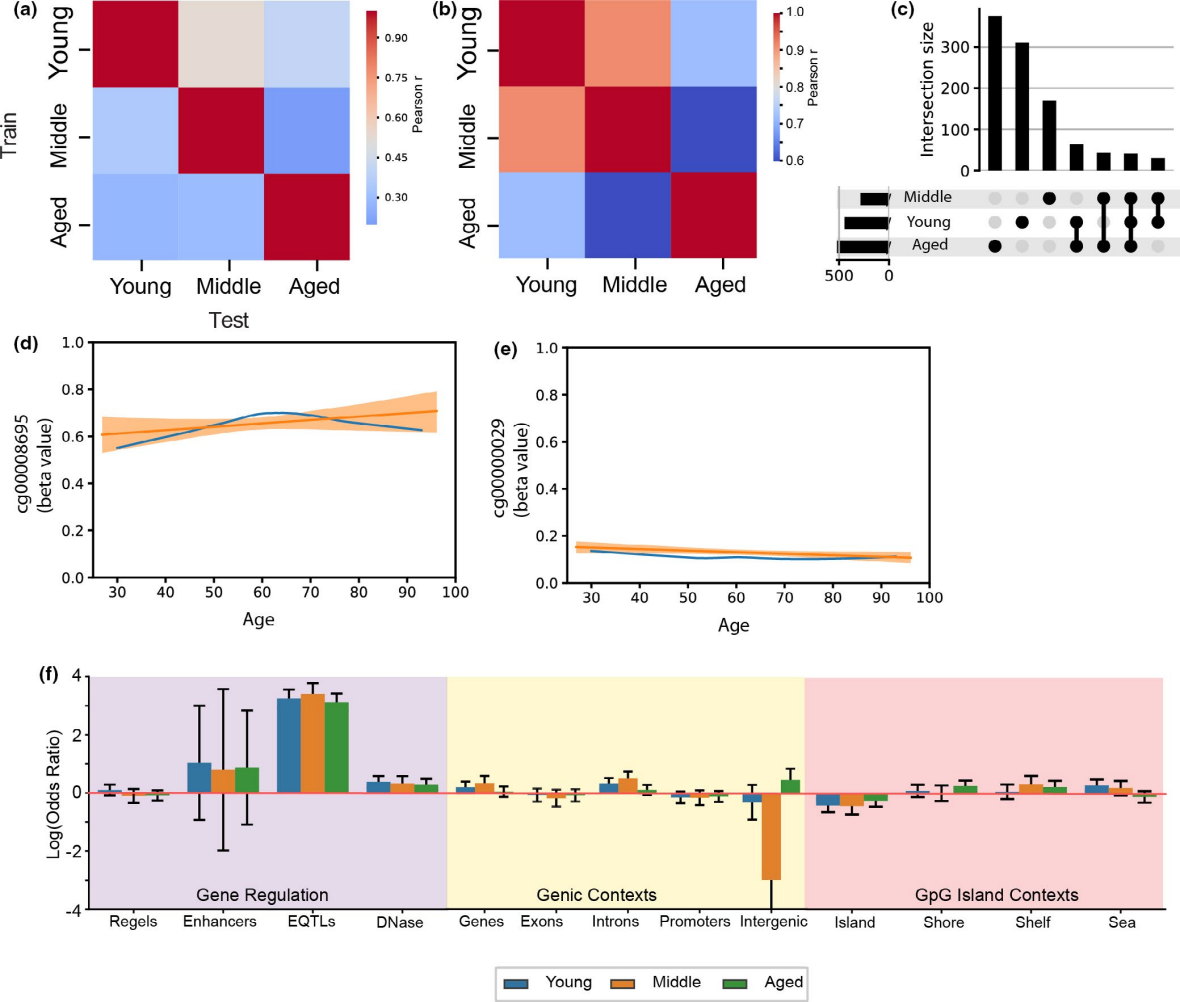
Clocks trained on discrete age groups show markedly poor performance at predicting higher/lower age groups. (a) Matrix of prediction accuracy from epigenetic clocks trained and tested on age three age bins. Each age bin represents a third of the ages from 25 to 100 (e.g., ‐ Young =ages 25–50). (b) Matrix of correlations between clock site coefficients common between clocks trained on Young, Middle, and Aged groups. (c) Upset plot showing the number of loci in each trained clock and their combinatorial overlaps. (d and e) Line plots of two example loci comparing loess regression (blue) to linear regression (orange) in the GSE60185 dataset. (d) a clock site with a parabolic distribution that reflects into a linear one. (e) a clock site where a linear model is a good fit for age‐related changes in methylation. (f) Bar plots of log odds ratios showing enrichment\depletion of loci from clocks with different training age range with respect to regulatory features, genic features, and CpG islands, corrected for genomic distribution of sites measured on the 450k array

We have made two key observations regarding methylation aging, namely 1) non‐linear site selection outperforms linear for training epigenetic clocks (Figure [Link], [Link], [Link]) and 2) age‐predictive loci have very small changes over the lifespan (Figure 1). This raised the question of how sites that would need to change by a fraction of a percent per year can consistently predict age. These small changes are observed even in a pan‐tissue context. The changes are much larger than we would expect from senescent cells, which are relatively rare and appear at different rates between tissues (Tuttle et al., 2020), and also are unlikely explained by some change in blood constituent cells (Chen et al., 2016) as they would vary wildly in their abundance with vascularity of the tissue, such as between whole blood and saliva. To answer this question, we performed an experiment to see if the most predictive loci from full age range models are identifiable in windows within the lifespan (Figure 4a). We trained three epigenetic clocks separately using data from three groups: young (25–50), middle (50–75), and aged (75–100) samples, then tested them on each age group. We found sites most predictive of aging in younger ages (25–50) were poor predictors later in life (75–100). Based on our observations of the linear and non‐linear relationships, we then looked at how clock sites from both Horvath and our own clocks change throughout the lifespan. We found that 99.5% of loci exhibit a distribution with at least one inflection point with aging that could be regressed into an overall linear trend (Figure 4d, Figure [Link], [Link], [Link]), alongside loci where locally fitted regression matched the sites’ linear fits (Figure 4e). While other reports (e.g., ‐ (Marioni et al., 2019)) have noted a non‐linear change with age, the parabolic distribution we observe appears to be novel. These changes may be attributable to changes in variance with aging (Slieker et al., 2016), but they describe an overall trend to increasing variance that would not necessarily lead to a parabolic distribution. Since these parabolic sites are also selected by epigenetic clocks on the basis of an extrapolated linear trend, further exploration of the trajectory of methylation aging may yield understanding of how clock predictions would respond to aging interventions and diseases. Similar to tissue‐specific clocks, clocks trained on discrete sections of the lifespan are largely uniformly distributed with an enrichment near eQTLs (Figure 4f).

### 2.5 Age‐predictive methylation loci are depleted in biologically informative regions

**FIGURE 5:**
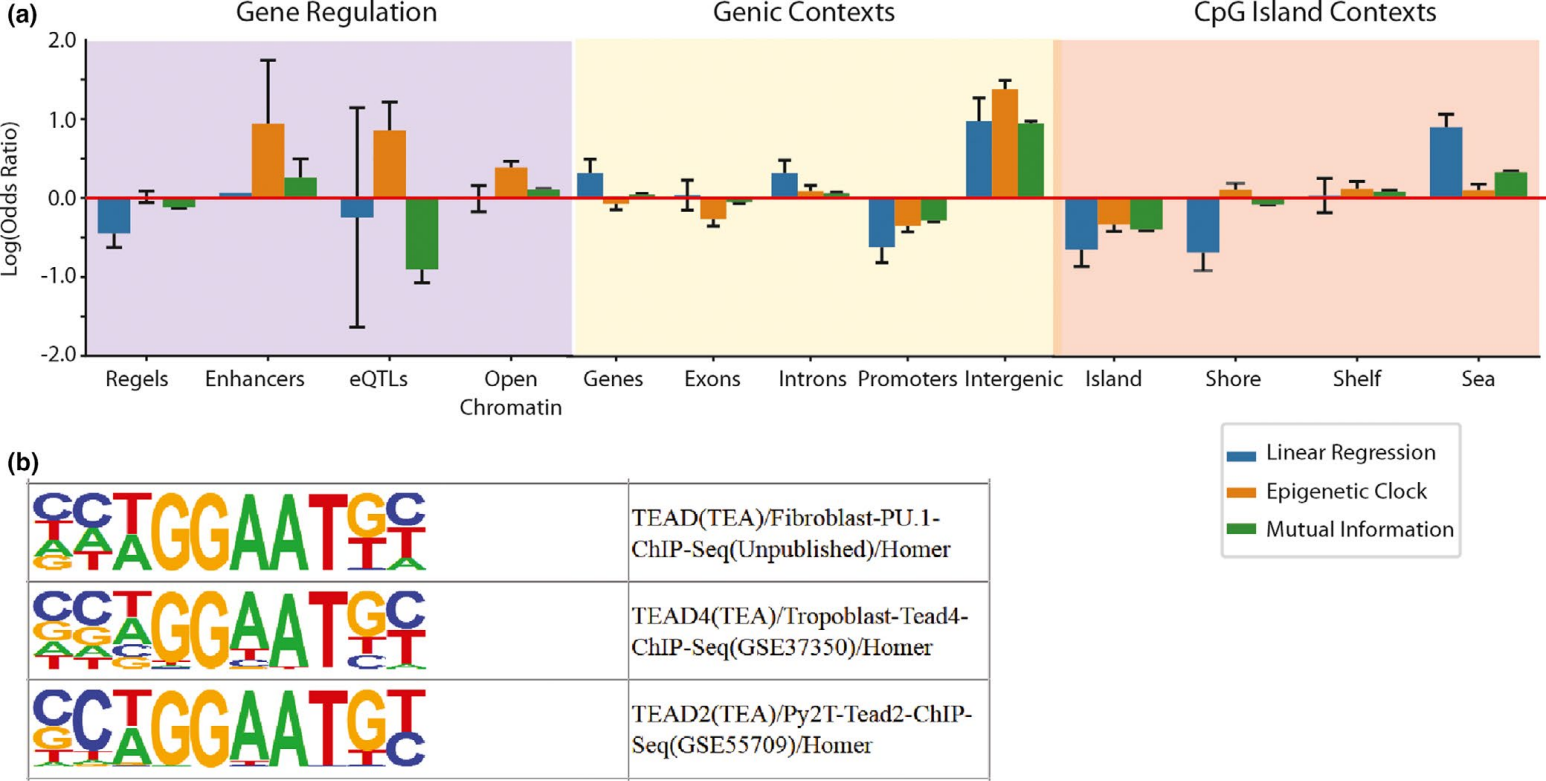
Genomic feature enrichments for age‐related loci. Epigenetic clock loci are enriched in information‐poor regions of the genome. The top quartile of mutual information loci (green), sites with significant age effects by OLS regression (blue), and sites chosen in an epigenetic clock built on the full 450k dataset (orange) was compared to known genomic features. (a) Bar plots of log odds ratios showing enrichment\depletion of loci from multiple models with respect to regulatory features, genic features, and CpG islands. Age‐related methylation loci are enriched in intergenic regions and depleted in gene promoters. For CpG islands, shores were defined as 2kb up and downstream of the CpG island body, and shelves were defined as 2kb up and downstream of the shores. Age‐related methylation loci are enriched in the open sea (outside of CpG islands) and depleted in the island bodies. Age‐related methylation loci selected by mutual information and regression are depleted in enhancers, eQTLs, and other regulatory elements (TFBS, repressors, etc.), and distributed evenly throughout euchromatin and heterochromatin. Epigenetic clock sites are however enriched in open chromatin, near eQTLs and gene enhancers. (b) Table with logos of most enriched motifs from HOMER analysis of the probe sequence from epigenetic clock sites

To understand the biological relevance of different loci that are predictive of or related to aging, we performed genomic enrichment analyses on three sets of methylation loci. These sets were as follows: 1) 6666 loci we determined that change with age by linear regression, 2) 2624 that serve as predictive clock sites, and 3) 79921 sites related to aging as measured by mutual information. When exploring the 6666 age‐related sites identified by traditional statistical analysis (linear regression) we found strong enrichments for genomic regions with limited known biological function for DNA methylation such as intergenic regions and sites outside of CpG islands, with under‐representation in regions such as promoters and CpG islands where DNA methylation is understood to regulate gene expression/genomic accessibility (Figure 5). The results were similar but weaker for age‐related sites selected using mutual information. In contrast, epigenetic clock sites were previously reported as enriched in VISTA (Visel et al., 2006) enhancers and near GTEX (Lonsdale et al., 2013) eQTLs. We attempted to further interrogate the activity state of promoters near age‐predictive cytosines, but it is impossible to generate a meaningful multi‐tissue prediction of activity with current data. Performing the enrichment against a target tissue of interest did not yield any significant results (Figure [Link], [Link], [Link]). They were also enriched in open chromatin regions as defined by aggregated DNase hypersensitivity data. However, the regulatory element enrichment appears to be driven by enhancers as aggregate regulatory elements, because subsets of enhancers, super enhancers, and repressors show neither enrichment nor depletion. All methods of identifying age‐related methylation sites found they were depleted in CpG island bodies and gene promoters while being enriched in intergenic regions.

We then analyzed the clock sites’ relationship to DNA‐binding proteins by comparing the probe sequence used to detect methylation loci to DNA binding motifs using HOMER. We attempted to interrogate the enrichments with respect to the “targeted” cytosines but found no significant results after multiple testing (Supplement [Link], [Link], [Link]). The top 3 most significant sequence enrichments for known DNA‐binding protein motifs were all TEAD proteins, which are enhancer binding proteins that aid in the initiation of transcription and have been linked to cellular senescence (Xie et al., 2013) and age‐related disease (Tsika et al., 2010). These enhancer enrichments were previously reported (Bell et al., 2019), but in their analysis were under the threshold for significance after adjusting for the array background. Nonetheless, these provide a potential mechanism to further explore the biological impact of altered clock site methylation.

### 2.6 Epigenetic Age Acceleration, Aging Diseases, and the Trajectory of Aging

**FIGURE 6:**
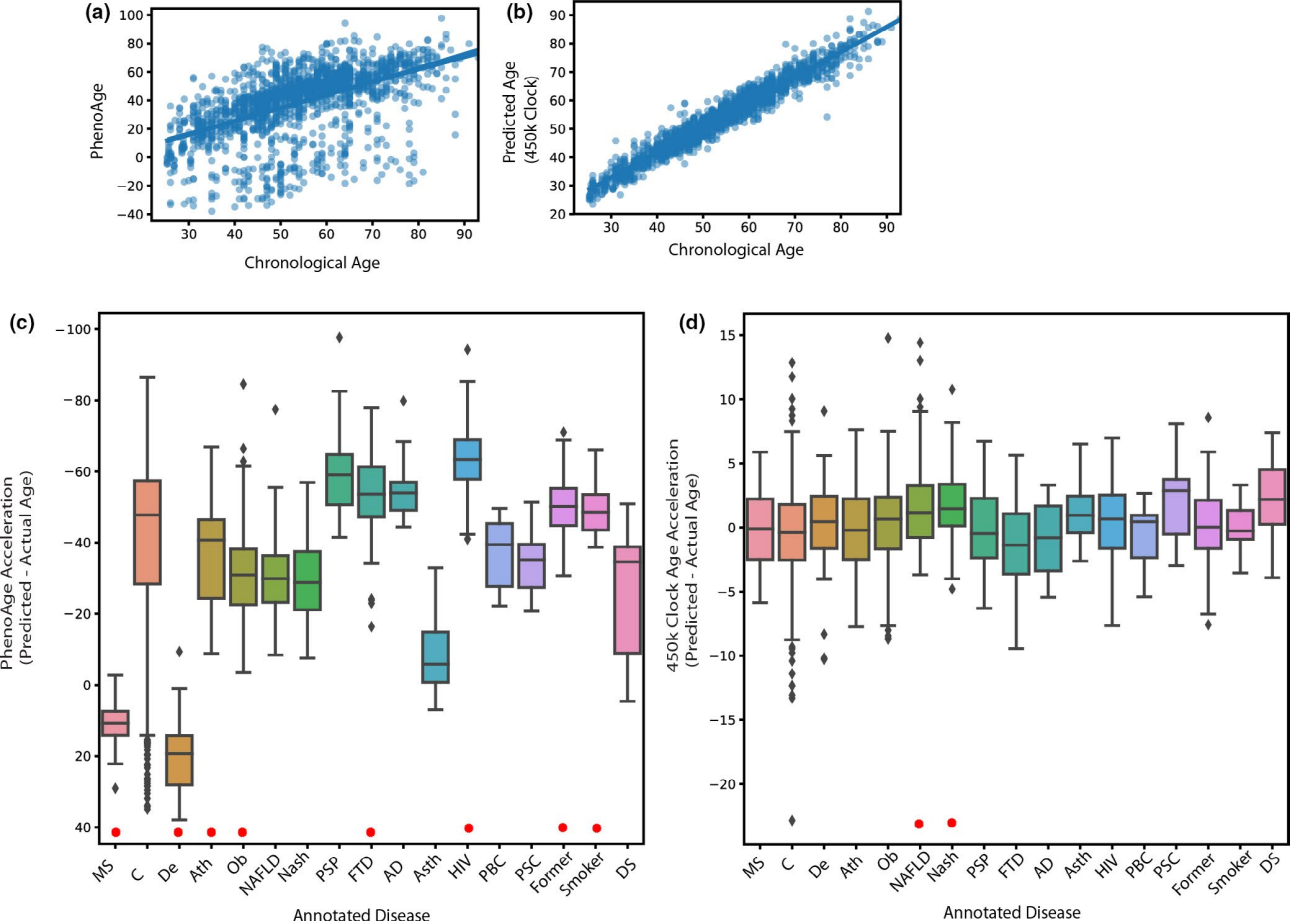
Epigenetic age acceleration associations from chronological and biological clocks across many age‐related states. We annotated 1767 samples for their control or potentially age‐related states. (a) PhenoAge predicted age vs chronological age in the disease‐annotated samples. (b), Our 450k clock predicted age vs chronological age. (b) and (c), Age acceleration distributions were compared using a one‐way linear model with holm‐adjusted t test post hoc tests. Significant differences (*p* <.05) from pooled healthy controls marked with a red asterisk (*). (c) Age acceleration as computed with PhenoAge (Levine et al., 2018).(d), Age acceleration computed with our 450k clock model. A table of the included experiments and their annotations can be found in the supplement (Supplemental S2). Abbreviations: MS—multiple sclerosis, C—aggregate controls from all included studies, De, depression; Ath, atherosclerosis; Ob, obese; NAFLD, nonalcoholic fatty liver disease; NASH, nonalcoholic steatohepatitis; PSP, progressive supranuclear palsy; FTD, fronto‐temporal dementia; AD, Alzheimer's disease; Asth, Asthma; HIV, human immunodeficiency virus; PBC, primary biliary cholangitis; PSC, primary sclerosing cholangitis; Former, former smoker; Smoker, current smoker; DS, Down's Syndrome

If epigenetic age acceleration is truly a biomarker of aging, we would expect samples from patients with age‐related diseases and conditions tend to have higher predicted ages than healthy samples. To this end, we annotated 1767 samples for a variety of conditions including multiple sclerosis, obesity, Alzheimer's disease, and smoking status (Figure 6). We compared measures of “age‐acceleration” in these groups using both our own chronological 450k clock and the published PhenoAge(Levine et al., 2018) “biological” clock. PhenoAge predicted most samples were age‐decelerated, with multiple sclerosis, obesity, and depression being significantly “accelerated” compared to controls (Figure 6a). Meanwhile, smoking status and HIV decreasing age acceleration seem contradictory to prior reports (Esteban‐Cantos et al., 2021; Levine et al., 2018). Since the PhenoAge clock is regressed against 10‐year mortality risk coerced into units of years, perhaps it is not surprising that even “healthy” control samples are predicted to be age‐accelerated. In contrast, our chronological 450k clock had much closer to zero average age acceleration. Notable exceptions are the significant increase in “age acceleration” of patients with NAFLD or NASH (Figure 6b). In both cases, these analyses have an additional uncertainty term in their age predictions that make interpreting age acceleration values difficult, with most samples falling within the range of model error. A potential explanation for age acceleration predictions going the opposite direction one might expect is found in looking at trajectory of aging methylation values over the lifespan. Namely, some show a non‐linear fit that is “hidden” to the linear elastic net regression (Figures S4 and S5). While not all epigenetic clock sites exhibit this non‐linear relationship, we noted in our primary feature selection that selecting sites with the best linear relationship reduced performance much more than selecting sites by non‐linear feature selection (Figure [Link], [Link], [Link]). This may explain why some age‐related diseases appear to be “decelerated” due to the same methylation values being present earlier and later in life.

## 3 DISCUSSION

Our meta‐analysis of the largest available age‐annotated methylation dataset to date found: 1) as much as one fifth of the measured cytosines contains age‐predictive methylation patterns; 2) tissues show largely similar aging patterns despite having methylated regions that define their identity; 3) epigenetic clock sites are enriched in intergenic regions, gene enhancers and sites near eQTLs and 4) are depleted in the regions generally thought to have the largest direct impact upon gene expression (e.g., CpG Islands and gene promoters); 5) patients with age‐correlated diseases did not appear significantly age‐accelerated according to the chronological epigenetic clock.

The fact that many different sites can be used to create an epigenetic clock with minimal impact on predictive performance argues against the idea that methylation changes are either programmed or individually important. Yet, because the clock is robustly predictive and age‐related methylation changes are mostly similar between tissues, this argues against entropy as a driving force. This could be reconciled by hypothesizing some genomic regions and/or features receive less methylation maintenance than others. Perhaps the changes occur in regions of the genome where they have no consequence, and instead, vary with absolute time such as in determining speciation time using pseudogene mutation rates. This “pseudomethylation” would be problematic for modeling aging biology, as they would likely not respond to aging intervention. Methylation maintenance mechanisms (e.g., DNMT1) serve as a counterbalance against entropy. However, if some genomic regions are less maintained than others, then we would expect the probability of a methylation state change with age to be correlated with the degree to which it is subject to methylation surveillance and maintenance. Because maintenance costs energy, it is reasonable to hypothesize the degree of maintenance correlates with the adverse impact an unregulated change in methylation would cause. If so, the probability a site's methylation will vary with age would inversely correlate with its impact on an organism's survival.

It is interesting that in spite of tissue aging interactions being rare in the age‐related differentially methylated loci (Figure 3A and B), training clocks on specific tissues selects loci that poorly predict other tissues (3C). This is further seen in the case study on age‐related neurological diseases, where the disease‐associated methylation sites are also rarely clock sites (Figure 6b). The tissue‐independent aging loci that are selected by clocks trained on multiple tissues are depleted in regions canonically associated with DNA methylation, namely promoters and CpG islands (Figure 5a), and simultaneously defining age acceleration using these clocks poorly predicts age‐related disease in our case study. Although combining the knowledge that these disease‐afflicted samples are predicted to be “younger” and the identification of non‐linear methylation changes in clock sites, perhaps these values of age acceleration are rather measuring proper compensation by the system. This would be consistent with the observation that clocks sites are significantly enriched in open chromatin and TEAD‐binding regions (TEAD requires co‐factors to act, plus literature‐mining analysis (Marioni et al., 2019) of TEAD‐binding regions near genes merely suggest TATA‐binding proteins as a commonality, which is also a general motif). Under this assumption, being epigenetically “younger” could be a mixture of those failing to compensate (and thus died and had tissue collected to be measured). This could also simultaneously make aging interventions show “decompensation” as they are no longer needing to respond to the pressures of aging and thus the samples would be predicted to be younger.

Given that methylation changes with age are robust across tissues, yet small in magnitude, leads the field to question whether the “ticking” that drives them is due to changes in cell population composition, such as a reduction of pluripotent stem cells or an increase in senescent cells within every tissue, or possibly high magnitude effects in rare cell populations (e.g., immune cells in the CNS compared to astrocytes/neurons). In either case, it is not clear whether the phenomenon driving ticking clock sites is due to healthy compensatory changes or deleterious drift toward age‐related fragility. To address the whole tissue versus individual cell type hypothesis, we are currently working on an aging study using mice with cell type‐specific markers that will allow whole‐genome sequencing from specific cell types. By comparing the mouse clocks’ predicted ages between different cell types, we hope to identify if the clock is indeed a pan‐tissue phenomenon or is affecting some subset of cells “contaminating” all tissues. We are also working on analyzing paired senescent and non‐senescent cells from the same patients to determine if senescent cells are driving the clock's predictive accuracy. These analyses will use BS‐Seq instead of methylation arrays, allowing us to simultaneously explore how robust our biological enrichments are when determined using whole‐genome sequencing data.

Our finding that the relationship between age‐related disease and age acceleration seems contradictory to other research (Fransquet et al., 2019; Levine et al., 2015). However, these prior publications show average age acceleration values for patients that are within the error of predictive accuracy (i.e., disease samples are “age accelerated” by less than the ~4 year error range of epigenetic clocks). While we cannot provide a resolution to this dilemma with the currently available data, it should act as a caution when evaluating “age‐acceleration” as a researcher using smaller subsets of samples, especially when the used clock is not trained on similar sample types with similar distributions of methylation values.

In summary, the predictive power of the epigenetic clock is robust, but such a large fraction of the genome can be used to predict, the magnitude of the changes is small, and these regions tend to be depleted near genes. This leads us to hypothesize that the pan‐tissue predictive loci are more likely to be molecularly “silent” methylation changes that accrue outside of strong regulatory regions due to entropy in methylation maintenance, which must be explored in the future studies. Furthermore, if current models inconsistently annotate patients with age‐related diseases as “age‐accelerated” and the confidence by which one can declare a sample age‐accelerated is small, this argues against the idea that epigenetic clocks can disentangle biological age from chronological age.

## 4 EXPERIMENTAL PROCEDURES

### 4.1 Data and Label Collection

Raw data were collected from NCBI’s Gene Expression Omnibus (GEO). For replicating Horvath's experiments, individual datasets were extracted and manually curated from metadata using geoquery (Davis & Meltzer, 2007). Of these, only 20 datasets were available and matched the given age distributions and sample numbers as reported in Horvath's original report (Horvath, 2013). These data make up dataset 1, used for the direct Horvath replication (Figure 2b, d) and the iterative trimming models (Figure 2e). Our second dataset consists of all publically available data from the Illumina 450k Human Methylation BeadChip Array that could be annotated by our label extraction program ALE (Giles et al., 2017), with accompanying metadata as presented in geometadb (Zhu et al., 2008). Sample labels for all samples’ sex, age, and tissue were extracted from text using our previously published tool ALE (Giles et al., 2017). We then exclude any samples with annotated ages under 25 for two reasons. First, we see aging as a process that begins post‐development, with human development ending around 25 years of age. Second, our annotation model is often incorrect about units, and as a result, our confidence in ages <25 is much lower than the rest of the lifespan due to common age labels with units of weeks/months falling in this range. Disease annotations were hand curated by reading their metadata and the metadata of the associated GSEs. A table of annotations can be found in Supplemental Data [Link], [Link], [Link].

### 4.2 Data Preprocessing

The replication dataset for Horvath's clock model was preprocessed using code from Horvath 2013 to ensure identical normalization and imputation. We also used the same age transformation as reported in Horvath 2013(Horvath, 2013). The full 450k data were imputed in a similar pipeline, using KNN imputation in sets of 120,000 probes with subsequent normalization. For our linear models, batch effect correction was performed using ComBat (Leek et al., 2012) to control for experiment ID after removing samples which did not fall in the beta distribution.

### 4.3 Feature Selection

Due to the large number of samples and probes included in the full 450k dataset, modeling could not be performed on the full dataset simultaneously. As such, we tested many feature selection pipelines in the smaller Horvath subset to determine their ability to preserve useful sites for age prediction (Figure [Link], [Link], [Link]). This led us to using mutual information as the primary feature selection method, from which we selected the top ~20% of sites and using those 79931 loci for all downstream analyses.

F‐regression and mutual information were performed using sklearn's(Fabian Pedregosa et al., 2011) implementation on one quarter of all loci at a time due to memory constraints. Top 10% delta mean and variance were computed by comparing the mean and variance differences for each locus between the young (25–35) and aged (65–100) sets of samples.

### 4.4 Statistical Analysis

Elastic net regression was utilized to generate clock models, as in Horvath 2013. Horvath's replication was performed using R’s glmnet(Friedman et al., 2009), while the full 450k model was performed using sci‐kit learn's ElasticNetCV (Fabian Pedregosa et al., 2011). In both cases, 10‐fold cross‐validation modeling was used with the L1 vs L2 selection parameter at 0.5 (elastic net), and the selectivity parameter adjusted based on cross‐validation.

Age‐related locus analysis was performed using OLS regression in the model methylation ~age + tissue +age:tissue. After FDR multiple testing correction, 676 loci were left for downstream analyses. PhenoAge predictions were determined as described in Levine, et. al. (Levine et al., 2018).

### 4.5 Genomic Feature and Motif Enrichment

Identifying gene, gene‐related, and CpG island‐related enrichments were performed by using bedtools to annotate sets of loci with features from UCSC. Feature enrichments were computed based on hypergeometric tests comparing the various groups with a 10% FDR correction for multiple testing.

Motif enrichments were performed using both HOMER (Heinz et al., 2010) and MEME (Machanick & Bailey, 2011). Fasta files were constructed with the 50 bp sequence of each probe on the Illumina 450k Array for all loci, and age‐related loci from mutual information, elastic net, and OLS regression models. These loci were then analyzed for known and *de novo* motifs, with enrichments calculated against the background of all sites on the array.

Identifying enrichments in genomic features were based on data from UCSC genome browser. CpG islands and genic regions were defined using UCSC’s annotated island bodies and genes, with promoters being 2kb upstream of the TSS and shore/shelves being defined as 2kb blocks up and downstream of the island body. Simultaneous comparison of all regulatory elements was performed using ORegAnno regulatory elements (Griffith et al., 2007). Vista enhancers were used to look specifically at known human enhancers (Visel et al., 2006). eQTLs were taken from GTEx (Lonsdale et al., 2013). Chromatin density was inferred using DNase hypersensitivity data from ENCODE DNase‐seq (Consortium & E.P., 2004).

## CONFLICT OF INTEREST

The authors declare no conflicts of interest.

## AUTHOR CONTRIBUTIONS

H.L.P, W.M.F., and J.D.W. conceived and designed the study. W.M.F., C.G., and J.D.W. supervised the study. H.L.P., C.A.B., X.R., and C.B.G. produced code for sample processing and analysis. H.L.P. and C.G. performed statistical analyses. H.L.P., W.M.F., and J.D.W. wrote the manuscript. All authors discussed the results and commented on the manuscript.

## Supporting information

Supproting Information 1

Supproting Information 2

Supproting Information 3

## Data Availability

The datasets analyzed in the current study are available in NCBI GEO. These datasets are all from GPL13534 and can be obtained from https://www.ncbi.nlm.nih.gov/geo/query/acc.cgi?acc=GPL13534.
