# Supplementary material for: Many chronological aging clocks can be found throughout the epigenome: Implications for quantifying biological aging
Source: Aging Cell. 2021 Oct 16;20(11):e13492. doi: 10.1111/acel.13492 (PMC8590098; doi:10.1111/acel.13492)
Supplement: Supplementary file 1 — Supproting Information 1 [file ACEL-20-e13492-s002.docx]

**Supplemental Material**


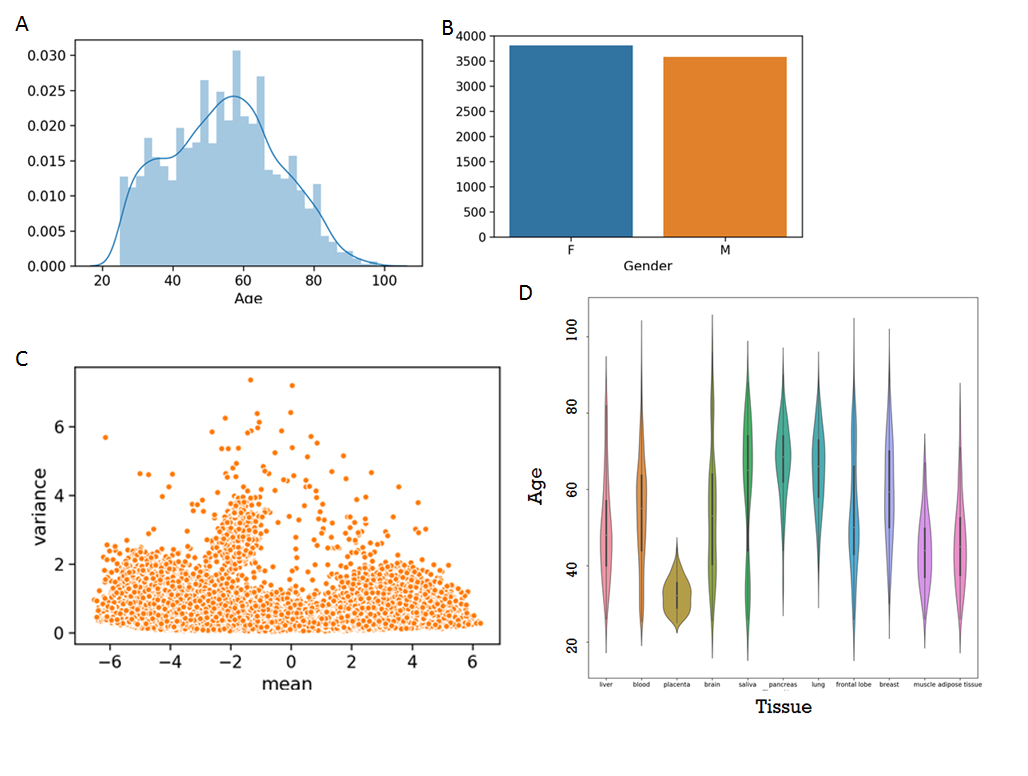


Supplemental Figure 1 – Descriptives

**A** Density plot of ages from all annotated 450k samples in our dataset

**B** Male and female sample numbers from annotated samples

**C** Mean vs variance after ComBAT normalization. Data represented as M-values to further show distribution.

**D** Violin plots of the top 10 most abundant tissues with given annotated age distributions


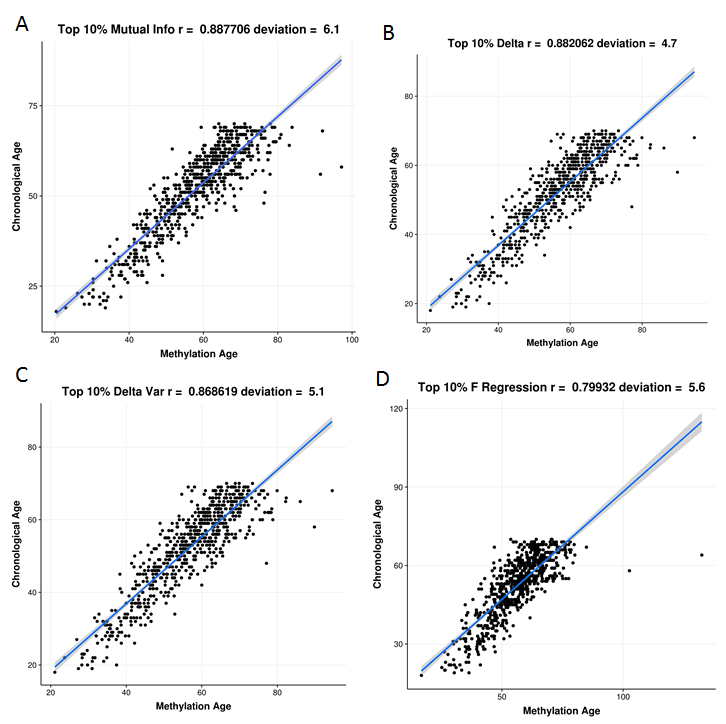


Supplemental Figure 2 – Testing primary feature selection on Horvath datasets

To determine how to best reduce the dimensionality of the large dataset from the Illumina 450k array, we tested primary feature selection in Horvath’s original ~21k training sites. Results for chronological vs methylation predicted age found that mutual information was the most accurate of the feature selection methods.
**A** Epigenetic clock trained using the top 10% most informative sites by mutual information regression
**B** Epigenetic clock trained using the top 10% most changed sites between young and old

**C** Epigenetic clock trained using the top 10% sites by highest variance

**D** Epigenetic clock trained using the top 10% of sites ranked by F regression


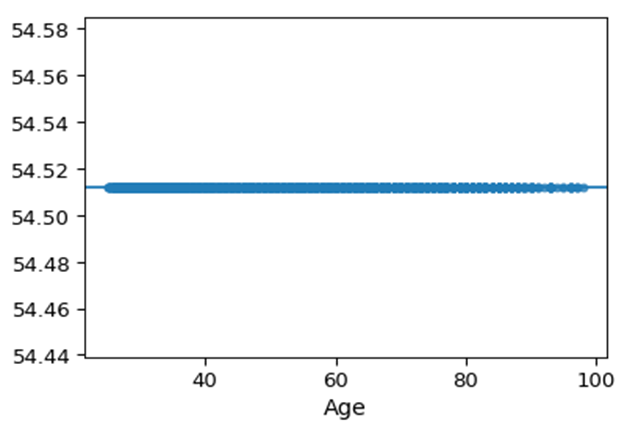


Supplemental Figure 3 – Dummy Regressor Model Age Predictions

A dummy regressor model that simply predicts the mean age value for all samples performs very poorly (r = -0.04, MAD 17.809 years, p > .05) compared to trained epigenetic clock models.


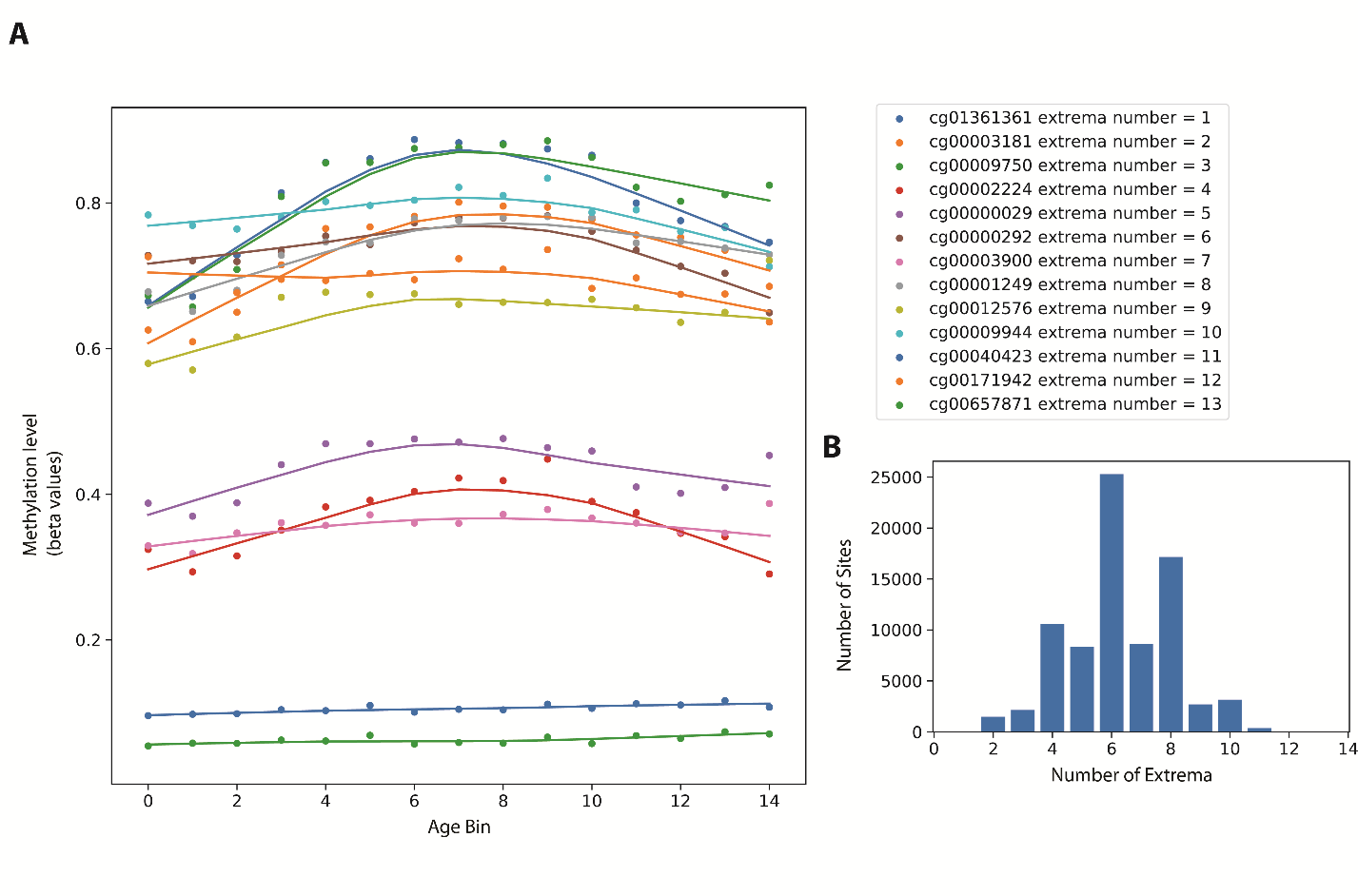


Supplemental Figure 4 – Quantifying Extrema to Identify Non-linear Age Associations

**A** Examples of 13 clock sites with varying numbers of extrema in their distribution throughout the lifespan **B** Barplot of extrema numbers for all sites in the epigenetic clock training set (79921 loci selected by mutual information)


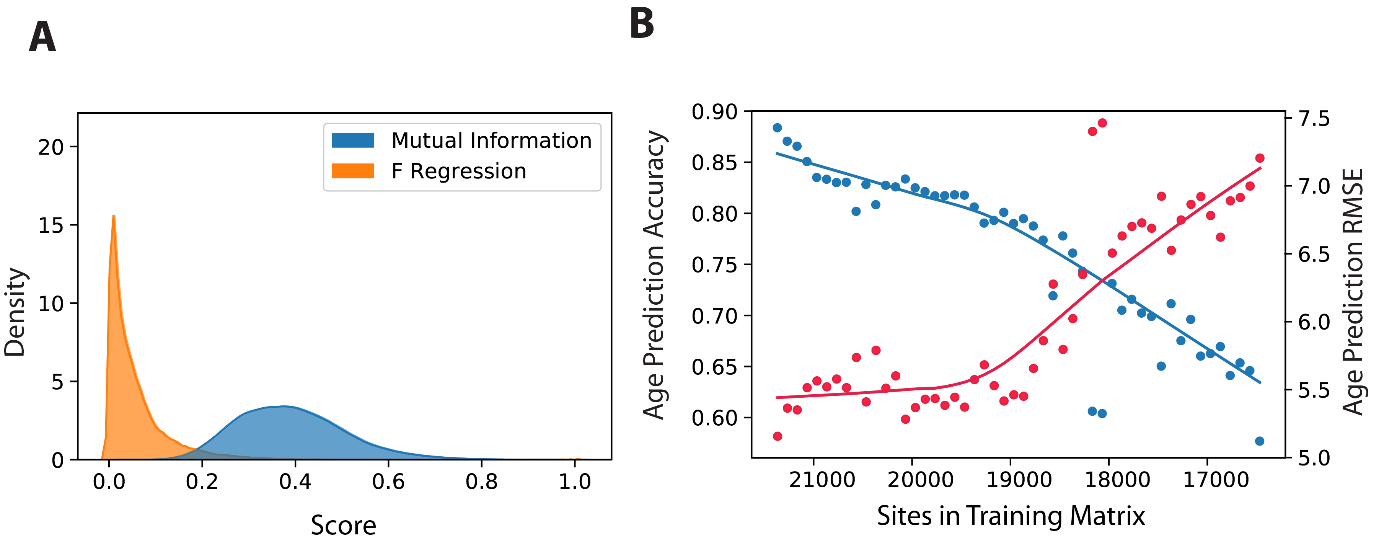


Supplemental Figure 5 – Comparison of Linear and Non-linear methylation-aging associations

**A** Density plot of weighted linear (F-test for linear regression) and weighted non-linear (mutual information) scores for age-associated methylation changes for all loci on Illumina 450k (scaled to 0=lowest age-association; 1=maximum age-association). The linear regression relationships show a strong skew with most loci having much smaller age-linked coefficients. Meanwhile, the mutual information scores are closer to normally distributed about a moderate mean weighted value (0.4) with some skew toward higher associations.


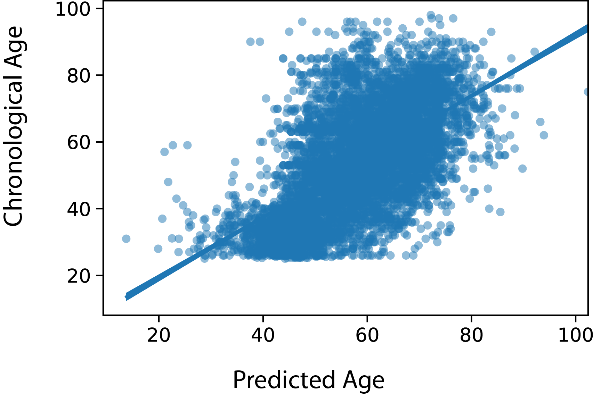


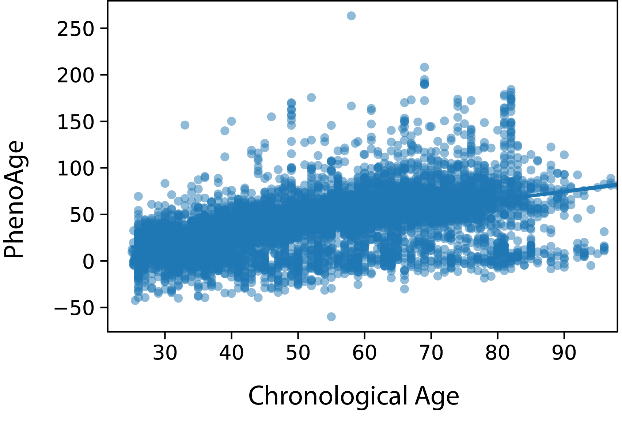
Supplemental Figure 6 – Correlations between predicted and actual age on all tissues from a clock trained on saliva.

Supplemental Figure 7 – PhenoAge vs Chronological Age.


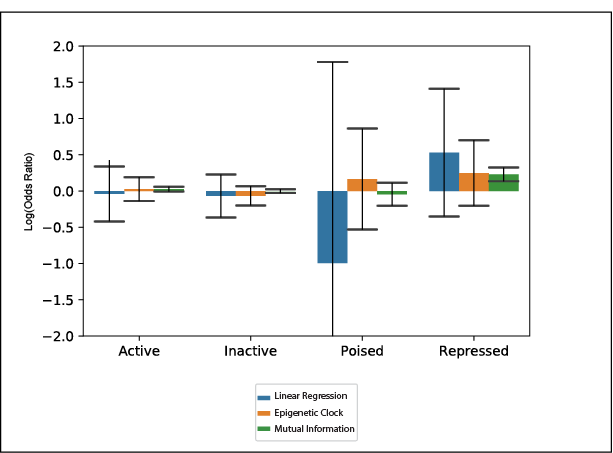
Supplemental Figure 8 – Genomic enrichments of mutual information and clock enrichments against neuron-derived promoter activity

Supplemental Table 1 – Genomic region overlaps with per-tissue clocks

Supplemental Table 2 – Genomic region overlaps with clocks trained on binned ages

Supplemental Table 3 – Genomic region overlaps from our chronological epigenetic clock compared to primary feature selection (mutual information regression) and simple linear regression.
